# ‘Uncrunching’ time: medical schools’ use of social media for faculty development

**DOI:** 10.3402/meo.v18i0.20995

**Published:** 2013-06-27

**Authors:** Peter S. Cahn, Emelia J. Benjamin, Christopher W. Shanahan

**Affiliations:** 1 Center for Interprofessional Studies and Innovation, MGH Institute of Health Professions, Boston, MA, USA; 2 Department of Medicine, School of Medicine, Boston University, Boston, MA, USA; 3 Department of Epidemiology, School of Public Health, Boston University, Boston, MA, USA

**Keywords:** faculty development, social networking, collaboration, Web 2.0, time demands

## Abstract

**Purpose:**

The difficulty of attracting attendance for in-person events is a problem common to all faculty development efforts. Social media holds the potential to disseminate information asynchronously while building a community through quick, easy-to-use formats. The authors sought to document creative uses of social media for faculty development in academic medical centers.

**Method:**

In December 2011, the first author (P.S.C.) examined the websites of all 154 accredited medical schools in the United States and Canada for pages relevant to faculty development. The most popular social media sites and searched for accounts maintained by faculty developers in academic medicine were also visited. Several months later, in February 2012, a second investigator (C.W.S.) validated these data via an independent review.

**Results:**

Twenty-two (22) medical schools (14.3%) employed at least one social media technology in support of faculty development. In total, 40 instances of social media tools were identified – the most popular platforms being Facebook (nine institutions), Twitter (eight institutions), and blogs (eight institutions). Four medical schools, in particular, have developed integrated strategies to engage faculty in online communities.

**Conclusions:**

Although relatively few medical schools have embraced social media to promote faculty development, the present range of such uses demonstrates the flexibility and affordability of the tools. The most popular tools incorporate well into faculty members’ existing use of technology and require minimal additional effort. Additional research into the benefits of engaging faculty through social media may help overcome hesitation to invest in new technologies.

Recognizing the importance of enhancing faculty members’ teaching skills and career advancement, academic medical centers have invested in faculty development (1, 2). Individual medical schools have established offices for faculty development and have launched comprehensive curricula to support faculty in their academic roles (3, 4). As opportunities to receive professional development have increased, however, so have work demands on faculty, making it difficult to take advantage of voluntary offerings. In surveys of faculty at institutions as disparate as McGill University (5), Maine Medical Center (6), and Catholic University of Chile (7), faculty members reveal that the most significant barrier to participation in faculty development activities is the lack of time.

Organizers of faculty development programs have experimented with using technology to reach faculty unable to participate in traditional, onsite lunchtime workshops. Children’s Hospital Boston, for example, distributes an electronic newsletter with summaries of seminars and even local restaurant reviews (8). Similarly, UC Davis allows faculty based outside the main campus to attend events virtually through teleconferencing (9), and many other faculty development offices post slides or recordings of past events on an external website. Unfortunately, these tools lack the potential for community building that comes with user-generated social media.

Use of social media, also called Web 2.0, has gained widespread acceptance. According to the Pew Research Center, in 2012, 67% of all Internet users in the United States participated in social networking, with an even higher rate among adults aged 50 years or younger (10). Of those active on social networks, 15% have received health information from peers online (11). Academic health centers have recognized this familiarity by using Twitter and Facebook to communicate with prospective students and patients (12, 13). Moreover, dynamic, online technologies have become staples of medical education (14, 15) and scientific collaboration (16). In addition to posting users’ reviews, MedEdPORTAL, an online repository of medical education documents, receives over 2,000 downloads a month of materials (17). As more academic physicians and scientists come from the ranks of Generation X (born 1965–1979) and the Millennials (born 1980–1999), faculty development leaders have the opportunity to use social media to promote interactive, asynchronous learning (18).

Employing social media for faculty development may resolve the difficulty many participants encounter in attending face-to-face events. With online communities, faculty can join at convenient times from any location and still feel connected. Because faculty development offices tend to direct their programs at internal audiences, their communication strategies often go unnoticed by colleagues at other institutions. To help all institutions overcome this time crunch, we sought to document the extent of social media use for faculty development by providing a broad overview as well as selected examples of innovative practices.

## Methods

In conducting this study, we first determined the relevant members of two domains (faculty development offices and social media) and then identified areas of overlap. Following the expansive approach of Clyde Evans, the former director of Harvard Medical School’s Office for Academic Careers, faculty development was defined ‘not merely as improving teaching skills, but more broadly as nurturing the growth of trainees into accomplished faculty’ (19). In this sense, faculty development initiatives included mentoring, skill-building seminars, one-on-one consultation, grant programs, orientations, and leadership training. Faculty development programs may also focus on enhancing clinical, research, pedagogical, or administrative competencies.

To assure comparability with other published studies of social media in academic medicine, we limited our investigation to the 154 accredited medical schools in the United States and Canada (20). One investigator (P.S.C.) conducted the initial search in December 2011 – starting from the home page of each institution and using the site’s internal search box to retrieve pages relevant to the keywords, ‘faculty development,’ ‘faculty affairs,’ ‘career advancement’, and ‘professional development.’ The first 10 results of each search were examined to determine if the site met the inclusion criteria:

1. Aimed at faculty members in a department, unit, or entire school of medicine.
2. Contained links related to improving faculty performance or academic advancement.

Because of the broad scope of faculty development, several different offices within the same institution may take responsibility for implementing related programs (1). In those cases, multiple web pages were examined.

Next, main navigation items of each home page meeting the inclusion criteria were recorded, and subsequent links to all subpages were examined. Specifically, the search was directed to the presence of social media features as defined by Kamel Boulos and Wheeler (21), including:

1. blogs
2. Facebook
3. Flickr
4. iTunesU
5. social bookmarking
6. Twitter
7. video sharing sites like Vimeo and YouTube
8. wikis

To be included, these tools had to be tailored to faculty development efforts, not ‘all purpose’ channels for communicating to multiple audiences. Finally, instances of particularly sophisticated or creative uses of social media were noted.

To cross-reference the findings, the first author visited social media sites directly and used the sites’ internal search engines to find relevant hits for the same four keywords. Results for sites related to academic medicine were then compiled to create a master table of all social media tools found (http://www.bumc.bu.edu/facdev-medicine/faculty-development-and-diversity-committee/faculty-development-websites/). This page contains links to all offices of faculty development at the schools examined and indicates where and which social media tools are used.

In February 2012, the last author (C.W.S.) independently revisited the data to ensure accuracy and to verify the longevity of the social media accounts, repeating the review protocol for all 154 accredited medical schools. After resolving discrepancies between the two sets of findings, the second author (E.J.B.) selected 10 institutions from the revised master table to verify the validity of the results. The Institutional Review Board at Boston University Medical Center approved the study as exempt.

## Results

All but two of the 154 medical schools host at least one website devoted to some aspect of faculty development. In repeating the search for social media tools on the medical schools’ websites, this finding was confirmed with three exceptions:

1. Two additional Facebook pages and two blogs were discovered;
2. One existing Twitter feed was disqualified as pertaining to clinical research (rather than faculty development); and
3. Two existing institutional iTunesU accounts were disqualified for not specifically addressing faculty development.

The first author concurred with these changes and adjusted the master table of results accordingly (see Table 1 in Appendix). Spot checks of 10 medical schools’ websites by the second author verified the joint findings.

**Fig. 1:**
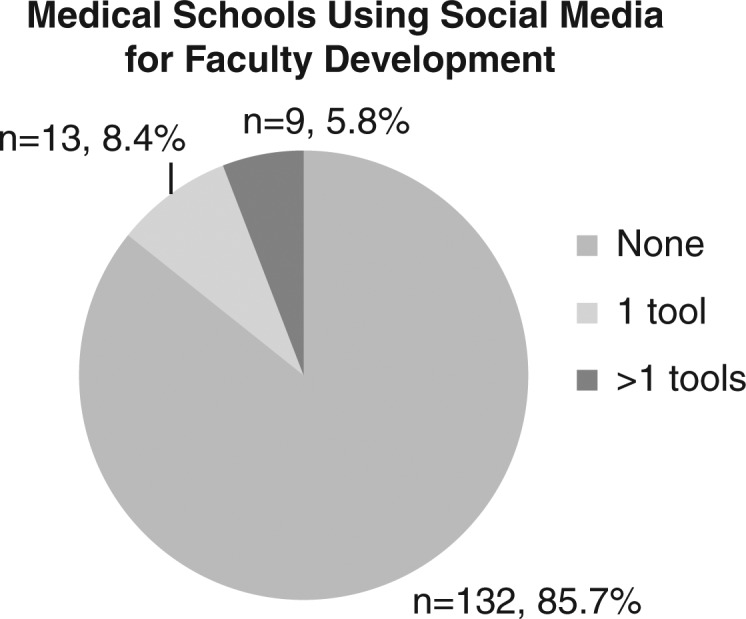
US and Canadian medical schools using social media for faculty development.

As part of their web presence, 22 medical schools employ at least one collaborative online tool aimed at faculty members (see Fig. 1). Across these 22 schools, 40 separate instances of social media were noted – the most popular being Facebook (nine institutions), Twitter (eight), and blogs (eight).

In particular, four faculty development programs are notable for their innovative and wide-reaching use of social media:

1. The University of Michigan Medical School structures its entire faculty development website as a wiki to invite user participation (22). YouTube videos explain how to use the site and feature interviews with faculty about their career progression. Users can comment on every menu item and calendar entry. The website’s designers have even developed a free application (‘app’) that delivers content optimized for mobile phones.
2. The University of California at San Francisco also takes an integrated approach. Its medical education Twitter feed appears on the school’s Medical Education blog. Conversations after faculty development workshops can continue on a collaborative workspace where materials and references are posted alongside a discussion board.
3. Duke University’s Office for Faculty Development uses its Facebook page to highlight relevant opportunities and articles, which appear in members’ individual news feeds. The events page allows the office to promote upcoming workshops and to show the roster of those who plan to attend. Virtual albums of photos highlight past events and invite comments.
4. The University of Saskatchewan supplements a roster of faculty development workshops with moderated discussion threads on a medical education wiki. Over 600 members have joined the wiki, where they can vote on which comments they find the most valuable. A blog highlights timely articles related to faculty development.

## Discussion

Among the faculty development offices that use social media, the most frequently employed tools are Facebook, Twitter, and blogs. The popularity of these sites reflects their overall use by health professionals. Facebook, for instance, serves as a platform for conducting epidemiological research, providing support to patients, and conveying health information (23). Twitter, a free microblogging service, is popular with physicians (24) – so it reaches faculty members where they may already be active. The tools used less frequently (e.g., wikis, iTunesU, Flickr, and Vimeo) tend to require more software- and hardware-related infrastructure. Contributing to wikis and watching videos of seminars also places additional time burdens on faculty; not surprisingly, the most popular social media platforms for faculty development allow users to assimilate information and voice opinions with just a few clicks.

While some medical schools have experimented creatively with social media to advance faculty development goals, the practice remains relatively uncommon. The slow adoption of social media for faculty development by medical training programs may result from several factors. First, using social media requires significant administrative effort and technical expertise to launch and maintain. Lacking any compelling evidence of impact, they may be less willing to keep the sites updated and functioning. Second, learning to navigate new web interfaces may impede some faculty from joining social networks. Toward this end, at least one study has documented a need for providing extensive training to new users (25). Finally, faculty development leaders may fear the potential for negative or inflammatory content to appear on unmoderated online channels.

Despite these barriers, institutions ranging from elite, private academic medical centers to publicly funded universities across all regions of the United States and Canada have embraced social media, demonstrating the flexibility and affordability of the technology. Although our survey did not measure either the number of users or the intensity of activity, free software can now monitor Internet traffic as well as engagement with content. To acquaint new users with the logistics of social media, faculty development offices should choose relatively simple and commonly used platforms, such as Facebook and Twitter. Just as business marketers have adapted to the loss of tight control over communications with networked customers, so must academic institutions accept the non-hierarchical flow of information on social media (26). In the end, one-to-one contact can promote interprofessional connections and community building. If necessary, medical schools may modify existing professionalism guidelines for student use of social media to create policies governing departmental and faculty online activity (27, 28).

## Limitations

This study has three primary limitations. First, due to the dynamic nature of online technologies and the varied rate of adoption by medical schools, the reliability of these data is somewhat problematic. Indeed, revisiting faculty development websites today might well yield several more examples of social media, not to mention the emergence of new sites (e.g., Google + ). Conversely, because of the rapid cycle of innovation, development, and failure of online tools, other examples of experiments in faculty development might have failed and disappeared before completion of this study.

Second, we reviewed the web presence only for faculty development offices housed in undergraduate training programs. Faculty members in academic medicine work in over 400 teaching hospitals and health systems, many with their own resources for faculty development. Faculty affiliated with Harvard Medical School, for instance, can affiliate with any of the eight offices devoted to faculty development and diversity. One of them, Children’s Hospital Boston, maintains an active Twitter account (@chbofd), but is not included in this study. Similarly, data collection did not capture items related to faculty development that reside on social networking channels controlled by an entire department or school.

Finally, the focus on social media also overlooks the burgeoning Internet technology of smart phone mobile apps, digital telephony like Skype, and RSS feeds that bypass the web browser altogether (29). Moreover, our methods capture only publicly available information. Admittedly, concerns about Internet privacy may restrict some forays into social networking to internal audiences; however, continued advances in this area will likely provide additional platforms from which to share related information.

## Conclusion

As much as medical faculty members value the opportunity to enhance their teaching, research, and professional skills, they face persistent limitations in their ability to participate in face-to-face programs. The scarcity of protected time away from clinical or educational responsibilities forces many faculty members to choose between income-generating activities and helpful but voluntary ones. Social media have the potential to alleviate some of the time crunch by delivering faculty development content through asynchronous, engaging channels. Particularly at a time when many physicians and scientists already use social media to communicate with peers, trainees, and patients, it makes sense that faculty development leaders could design programs that exploit online platforms.

Because of distinctive curricula and promotion criteria, most faculty development offices tend to target internal audiences. While understandable, such restrictions make creative solutions to common problems like advancement for clinician educators and revitalization for mid-career faculty members less accessible to peers at other institutions. As demands for faculty productivity increase, the difficulty of attracting attendance for in-person faculty development events will only become more acute; the use of social media could help ease this time crunch. By exploring both the range of interactive resources and current exemplars of social media use, perhaps more medical training programs will be encouraged to experiment with the growing number of online tools to promote faculty development.

## Ethical approval

Approved as exempt by the Boston University Medical Center Institutional Review Board, Protocol H-31008.

## Previous presentations

‘Untangling the Web: Innovative Strategies for Engaging Faculty Online’, Workshop presented at the 2011 AAMC Group on Faculty Affairs Professional Development Conference, Seattle, WA.

## Appendix

**Table 1:** US and Canadian medical schools’ use of specific social media for faculty development

Institution	Twitter	Facebook	YouTube	Blog	Wiki	iTunesU	Flickr	Vimeo
Boston University	X	X		X			X	
Creighton University			X					
Dalhousie University	X			X				
Duke University		X				X		
Johns Hopkins University		X						
Medical College of Wisconsin	X	X						
Memorial University of Newfoundland	X							
Northwestern University		X	X	X				X
Stanford University		X						
Tulane University	X	X	X					
University of Alberta					X			
University of British Columbia			X					
University of California, San Francisco	X			X	X		X	
University of Colorado			X					
University of Maryland			X					
University of Michigan			X	X	X			
University of North Carolina		X						
University of Puerto Rico				X				
University of Saskatchewan	X			X	X			
University of Toronto				X				
Wayne State University	X							
Yale University		X						

## References

[1] Morahan PS, Gold JS, Bickel J (2002). Status of faculty affairs and faculty development offices in U.S. medical schools. Acad Med.

[2] Sambunjak D, Straus SE, Marusić A (2006). Mentoring in academic medicine: a systematic review. JAMA.

[3] Bland CJ, Seaquist E, Pacala JT, Center B, Finstad D (2002). One school’s strategy to assess and improve the vitality of its faculty. Acad Med.

[4] Newland MC, Newland JR, Steele DJ, Lough DR, Mccurdy FA (2003). Experience with a program of faculty development. Med Teach.

[5] Steinert Y, McLeod PJ, Boillat M, Meterissian S, Elizov M, Macdonald ME (2009). Faculty development: a “field of dreams”?. Med Educ.

[6] Trowbridge R, Bates P (2008). A successful approach to faculty development at an independent academic medical center. Med Teach.

[7] Montero L, Triviño X, Sirhan M, Moore P, Leiva L (2012). Barriers for faculty development in medical education: a qualitative study. Rev Med Chil.

[8] Emans SJ, Goldberg CT, Milstein ME, Dobriner J (2008). Creating a faculty development office in an academic pediatric hospital: challenges and successes. Pediatrics.

[9] Howell LP, Servis G, Bonham A (2005). Multigenerational challenges in academic medicine: UC Davis’s responses. Acad Med.

[10] Duggan M, Brenner J (2013). The demographics of social media users – 2012.

[11] Fox S (2011). The social life of health information, 2011.

[12] Kind T, Genrich G, Sodhi A, Chretien KC (2010). Social media policies at US medical schools. Med Educ Online.

[13] Giustini D (2006). How Web 2.0 is changing medicine. Br Med J.

[14] Wiecha J, Heyden R, Sternthal E, Merialdi M (2010). Learning in a virtual world: experience with using second life for medical education. J Med Internet Res.

[15] Robin BR, McNeil SG, Cook DA, Agarwal KL, Singhal GR (2011). Preparing for the changing role of instructional technologies in medical education. Acad Med.

[16] Sagotsky JA, Zhang L, Wang Z, Martin S, Deisboeck TS (2008). Life sciences and the web: a new era for collaboration. Mol Syst Biol.

[17] Novinskie E, Hunt S, Simpson C, Nash J (2012). MedEdPORTAL: advancing scholarship, sharing innovations, promoting continuing education.

[18] Bickel J, Brown AJ (2005). Generation X: implications for faculty recruitment and development in academic health centers. Acad Med.

[19] Evans CH (1995). Faculty development in a changing academic environment. Acad Med.

[20] Association of American Medical Colleges (2011). AAMC member medical schools.

[21] Kamel Boulos MN, Wheeler S (2007). The emerging Web 2.0 social software: an enabling suite of sociable technologies in health and health care education. Health Info Libr J.

[22] University of Michigan Medical School (2011). Faculty Career Development. http://www.webcitation.org/68Mw0bwNW.

[23] George DR (2011). “Friending Facebook?” A minicourse on the use of social media by health professionals. J Contin Educ Health Prof.

[24] Chretien KC, Azar J, Kind T (2011). Physicians on Twitter. JAMA.

[25] Quaas-Berryman F (2010). Faculty development 2.0: social networking tools as a means to enhance faculty inquiry.

[26] Li C, Bernoff J (2011). Groundswell: winning in a world transformed by social technologies. Expanded and revised ed.

[27] Landman MP, Shelton J, Kauffmann RM, Dattilo JB (2010). Guidelines for maintaining a professional compass in the era of social networking. J Surg Educ.

[28] Thompson LA, Dawson K, Ferdig R, Black EW, Boyer J, Coutts J (2008). The intersection of online social networking with medical professionalism. J Gen Intern Med.

[29] Anderson C, Wolff M (2010). The web is dead. Long live the internet. Wired Mag.

